# Influences of Cognitive Processing Capacities on Speech Perception in Young Adults

**DOI:** 10.3389/fpsyg.2017.00266

**Published:** 2017-02-24

**Authors:** Lily Tao, Marcus Taft

**Affiliations:** ^1^Institute of Cognitive Neuroscience, East China Normal UniversityShanghai, China; ^2^School of Psychology, University of New South WalesSydney, NSW, Australia

**Keywords:** speech perception, accent perception, executive functions, information processing speed, working memory

## Abstract

Foreign accent in speech often presents listeners with challenging listening conditions. Consequently, listeners may need to draw on additional cognitive resources in order to perceive and comprehend such speech. Previous research has shown that, for older adults, executive functions predicted perception of speech material spoken in a novel, artificially created (and therefore unfamiliar) accent. The present study investigates the influences of executive functions, information processing speed, and working memory on perception of unfamiliar foreign accented speech, in healthy young adults. The results showed that the executive processes of inhibition and switching, as well as information processing speed predict response times to both accented and standard sentence stimuli, while inhibition and information processing speed predict speed of responding to accented word stimuli. Inhibition and switching further predict accuracy in responding to accented word and standard sentence stimuli that has increased processing demand (i.e., nonwords and sentences with unexpected semantic content). These findings suggest that stronger abilities in aspects of cognitive functioning may be helpful for matching variable pronunciations of speech sounds to stored representations, for example by being able to manage the activation of incorrect competing representations and shifting to other possible matches.

## Introduction

In everyday communication, listeners encounter a multitude of variations in the speech signal, even from a single speaker, which can affect intelligibility. Accents in speech, both foreign and regional, are a source of variability that is commonly encountered, and one that can be particularly detrimental to speech perception. Indeed, accented speech has often been shown to produce speech processing costs (see e.g., Floccia et al., 2006; Adank et al., 2009; see Cristia et al., 2012, for a review). Understanding spoken language requires mapping acoustic input onto stored phonological and lexical representations. Therefore, during speech perception, listeners need to be flexible in accommodating acoustic signals that do not match perfectly with stored representations. When the mismatches are small, listeners are able to comprehend speech with minimal effort. When the variations are more substantial, however, such as with foreign-accented speech, additional cognitive resources may be required to process the speech signal (Van Engen and Peelle, 2014).

The present study investigated the potential influences of cognitive processing capacities on perception of accented speech. Specifically, executive functions, information processing speed, and working memory were assessed. For executive functions, there are varying definitions and frameworks of its components. One widely accepted account (Miyake et al., 2000) identifies three key components, namely inhibitory control, task switching, and updating. The present study will focus on the inhibition and switching components. Previous research (Adank and Janse, 2010) has shown that executive functions, particularly task switching, as measured by the Trail Making Test (TMT; which involves connecting dots in alphanumerical sequence, alternating between letters and numbers), predicts relative difficulty in understanding accented speech in a group of older adults, whereas information processing speed, as measured by the Coding test (which involves using a key to copy a series of abstract symbols paired with numerical digits), does not. The accent was an artificially created accent of Dutch, produced by reading from an adapted orthography. Adank and Janse (2010) also investigated perception of the novel accent by young adults, and found that they had significantly less difficulty in understanding the accent than the older adults. The measures of cognitive processing capacities, however, were not examined in those young adults. The present study, therefore, sought to investigate whether measures of cognitive processing capacities predict performance in perception of unfamiliar accents by young adults. When perceiving unfamiliar accented speech, older adults, given their declining cognitive functioning, may need to draw on mental resources to a greater extent than young adults (Pichora-Fuller, 2003; Adank et al., 2009). Nevertheless, cognitive processing capacities may still play a role in the perception of unfamiliar accents for young adults, and influence their performance on the speech perception tasks in the present study. In addition, a range of cognitive capacities were assessed, including two components of executive functioning, information processing speed, as well as working memory.

When processing accented speech, cognitive flexibility (i.e., “task switching” or “set-shifting”) may be required to match nonstandard pronunciations of words and speech sounds to stored standard representations. Furthermore, particularly when the accented pronunciations deviate more substantially from standard pronunciations, inhibitory control may be required to manage the activation of incorrect competing representations in order to make the correct match (Gordon-Salant and Fitzgibbons, 2004; Rudner and Lunner, 2014; Rönnberg et al., 2016). Speed of information processing, although not found to significantly predict older adults' performance in the understanding of a novel accent, may still be relevant to accent perception, as it may relate to the ability to rapidly pick up the regularities in the ways phonemes or speech sounds are replaced in accented speech (Pichora-Fuller, 2003; Adank and Janse, 2010). Therefore, the present study assessed both executive functions (including both switching and inhibition) and speed of information processing, to determine their influence on speech perception.

Working memory capacity was also assessed (using the Digit Span task, consisting of three parts—Forward, Backward, and Sequencing), since working memory has been found to influence the perception of speech, especially when of low intelligibility, such as speech-in-noise, speech produced by impaired speakers, or speech produced by a speech synthesizer (e.g., Francis and Nusbaum, 2009; Janse and Adank, 2012; see Akeroyd, 2008, for a review). Furthermore, by limiting the availability of working memory, such as by using a concurrent working memory task, speed and accuracy in the recognition of synthetic speech have been shown to be reduced, especially for low intelligibility synthetic speech (Francis and Nusbaum, 2009). The Ease of Language Understanding (ELU) model (Rönnberg, 2003; Rönnberg et al., 2008, 2013) describes the role of working memory in a wide range of speech listening conditions. In particular, if the phonological information extracted from the speech signal does not match with stored phonological representations (such as in speech-in-noise, distorted speech, or other low intelligibility speech), lexical access is then delayed, which in turn slows comprehension. Foreign accents in speech, particularly unfamiliar foreign accents, reduce intelligibility (Munro and Derwing, 1995; van Wijngaarden et al., 2002; Bradlow et al., 2010). Thus, working memory capacity may serve as a limiting factor for performance in the perception of accented speech.

Previous research has found influences of cognitive functioning on perceptual learning or adaptation to accented speech following exposure or training (Janse and Adank, 2012; Banks et al., 2015), as well as on perception performance with other types of nonstandard speech, such as speech-in-noise (Ellis et al., 2015), frequency-compressed speech (Ellis and Munro, 2013), and dysarthric speech (Baese-Berk et al., 2015). Many studies have shown that listeners can adapt to accented speech following even brief training exposures (e.g., Clarke and Garrett, 2004; Bradlow and Bent, 2008; Sidaras et al., 2009; see Samuel and Kraljic, 2009; Cristia et al., 2012, for reviews), and this adaption has further been linked with executive functions, particularly inhibitory control. Specifically, healthy young adults who performed better on the Stroop test adapted more and at a faster rate to a novel artificially created accent (Banks et al., 2015), while performance on a variation of the Flanker task was found to predict amount of adaption to an artificially created novel accent in older adults (Janse and Adank, 2012).

In regard to cognitive influences on nonstandard speech perception, Ellis and Munro (2013) showed that performance on the TMT and the Reading Span Test (RST; a measure of working memory span) were correlated with the recognition of speech-in-noise in a group of healthy adults. Ellis et al. (2015) further showed that performance on the TMT predicted recognition of frequency-compressed speech-in-noise, in both adults with and without hearing loss. TMT performance significantly predicted such speech recognition even when age (which was the strongest predictor found in that study) was partialled out. Using a different measure of task switching, namely the Intra-Extra-Dimensional Set Shift (IED), Baese-Berk et al. (2015) found that switching correlated with perception of dysarthric speech, as well as with perception of foreign-accented (Spanish-accented English) and regional-accented speech (Irish-accented English), in a group of healthy adults. However, inhibition, as measured by the Flanker task, did not have a significant influence.

The present study further investigated the influences of switching and inhibition on the perception of foreign-accented speech, in a group of healthy young adults. Unlike the study by Baese-Berk et al. (2015), this study examined multiple unfamiliar foreign accents. These were accents that would not be commonly heard in the participants' day-to-day environment, unlike Spanish- and Irish-accented English, which are comparatively more commonly heard in the USA where that study was conducted. A decrease in familiarity may lead to an increased reliance on cognitive resources for successful perception. Further, having multiple accents helps to ensure that accent perception is not influenced by relative familiarity with any particular accent. The present study also assessed other cognitive processing capacities, namely information processing speed and working memory, which may also have influences on the perception of unfamiliar foreign-accented speech, as outlined above.

## Methods

### Participants

There were 132 participants in this study (85 females). The mean age was 19.4 years (*SD* = 2.6), ranging from 17 to 33 years. All participants were either born in Australia or had arrived at or before age 1, and were raised and educated in Australia (i.e., had not spent a total of 1 year or more in another country). None of the participants reported having any hearing or speech impairments. The participants were students undertaking a first year undergraduate psychology course, recruited via the online participant recruitment system provided by the School of Psychology at the University of New South Wales (UNSW). They received course credit in exchange for participation.

### Stimuli/materials

#### Speech perception

Auditory stimuli used in this study were recorded using a Redback C0384 microphone onto a desktop personal computer. Each item (sentence or word) then had the beginning and end trimmed at zero crossings (trimmed on or as closely as possible to the onset and offset of initial and final speech sounds) using Audacity audio editor software.

An auditory sentence verification task was used to examine performance in the perception of both foreign-accented and non-foreign-accented speech. The task allows for assessment of the understanding of spoken statements, rather than only the recognition of strings of spoken words, as participants need to understand the sentences as a whole in order to make a response. Previous research has used such a task to assess processing deficits of unfamiliar accented speech (Adank et al., 2009), and perceptual adaptation to such speech (Janse and Adank, 2012). In the present study, the task involved an equal number of obviously true (e.g., “Birds have feathers.”) and obviously false statements (e.g., “Cats can lay eggs.”). The stimuli were taken from the “Silly Sentences” task (Baddeley et al., 1995; May et al., 2001), which was adapted from the Speed of Comprehension subtest of the Speed and Capacity of Language Processing (SCOLP) test (Baddeley et al., 1992). Part 1 of the task involved a set of 20 statements (10 true and 10 false), spoken in different foreign accents. Five speakers, each with a different uncommon foreign accent, were recruited to record the sentences. Effort was made in selecting speakers with uncommon accents that the participants were not likely to be familiar with. The accents were Danish (female), Jamaican (female), Mauritian (female), Russian-Hebrew (male), and Swiss German (male). The speakers were recruited through advertisements in the weekly International Student Forum newsletter, run by the Student Development International office at UNSW, and received $20 for their time. Part 2 of the task involved another set of 20 statements (10 true and 10 false), spoken in standard Australian English by five native monolingual English speakers (also three female and two male) who were recruited from the student pool undertaking a first year undergraduate psychology course at UNSW. The number of statements was evenly distributed among the five speakers in each part (i.e., four statements per speaker, with two true and two false). There were also six accented practice items (three true and three false), spoken by one speaker of Farsi (Persian) from Iran (female) who was not included as a speaker for any of the test items (see Appendix A in Supplementary Material for a full list of sentences used in this task).

To further examine processing of accented speech, an accented auditory lexical decision task was also used, which consisted of 40 words and 40 nonwords, plus 20 practice items (10 words and 10 nonwords). Processing of accented speech with single-word utterances may likely be more difficult than that with sentence stimuli, because there are fewer contextual cues to help identify the stimulus and fewer points of exposure within each trial for listeners to adapt to. Accented lexical decision tasks have been used in a number of previous studies examining the processing cost of an unfamiliar accent (Floccia et al., 2006, 2009), perceptual adaptation to an artificially created accent (Maye et al., 2008), and effects of training or exposure to phonemic category variations on subsequent identification of trained words (Norris et al., 2003) and novel words (McQueen et al., 2006). There were an equal number of monosyllabic and polysyllabic words (e.g., “score,” “discover”), and an equal number of monosyllabic and polysyllabic nonwords (e.g., “chusk,” “omsify”). The words were of moderate frequency of occurrence (ranging from 50 to 60 occurrences per million words, according to Carroll et al., 1971). Plural and past tense forms were avoided, as were conjunctions and prepositions. The nonwords were created so that they resembled actual words and were pronounceable. The same five accented speakers who produced the sentences for the auditory sentence verification task were asked to record the items in this task. The number of items was evenly distributed among the five speakers (i.e., 16 per speaker, with eight words and eight nonwords). The 20 practice items (10 words and 10 nonwords) were spoken by the same speaker who produced the practice sentences for the auditory sentence verification task (see Appendix B in Supplementary Material for a full list of items used in this task).

#### Executive functions

To assess executive functions, the TMT (Reitan, 1992), as was used in the study by Adank and Janse (2010), was first administered. The TMT focuses on the task switching component of executive functions, and was composed of two parts. Part A involved participants connecting numbered circles on a page, beginning with the number 1 and proceeding in numerical sequence up to 25. Part B also involved connecting circles in sequence, but alternated between numbers and letters (i.e., 1 to A, A to 2, 2 to B, and so on).

A second task assessing executive functions was also administered, namely the Color-Word Interference Test (CWIT) of the Delis-Kaplan Executive Function System battery (D-KEFS; Delis et al., 2001). The CWIT assesses both inhibition and switching, using the Stroop paradigm originally developed by Stroop (1935). This task consisted of four conditions. The two baseline conditions were Color Naming and Word Reading, which assessed key component skills of the other two higher level tasks, namely basic naming of color patches and basic reading of color words printed in black ink. The third condition, Inhibition, was the traditional Stroop task, in which participants needed to inhibit reading the words in order to name the incongruent ink colors (e.g., say “red” in response to the written word *blue* printed in red ink). In addition, there was a fourth condition, Inhibition/Switching, which required participants to switch back and forth between naming the incongruent ink colors and reading the words as indicated by a cue (a box surrounding the word). It has been shown that, by simultaneously requiring both inhibition and cognitive switching, the demands on executive functioning are greater in this condition than in the traditional Stroop Inhibition condition (Fine et al., 2008).

#### Information processing speed

Information processing speed was assessed using the Coding subtest from WAIS-IV (Wechsler, 2008), as was used by Adank and Janse (2010). The task has been shown to be a valid measure of processing speed, with performance on this task being primarily determined by the speed at which participants can process the necessary information. Other cognitive factors, like memory, may play a small role but do not contribute significantly to Coding performance (Crowe et al., 1999; Joy et al., 2004). The Coding task involved using a key to copy a series of symbols paired with numbers. The symbols resembled alphabetic letters, and involved one to two strokes to draw (e.g., ⊣, ⊢, ||).

A second information processing speed task was also included, namely the Alphabet Backwards task (Williams et al., 1996). The task has been found to be a brief but valid alternative to other more complex and time-consuming tests of information processing speed, and there is research providing support for its construct and discriminant validity (Williams et al., 1996). To perform this task, participants simply recited the English alphabet in reverse order (from Z to A).

#### Working memory

The Digit Span subtest from WAIS-IV (Wechsler, 2008) was used to assess working memory, which consisted of three parts—Forward, Backward, and Sequencing. Two of the three parts (Backward and Sequencing) involved simultaneously storing and manipulating information, which is a key aspect of working memory capacity (see e.g., Rönnberg et al., 2013; Wilhelm et al., 2013). Each of the three parts comprised 16 test trials, with every two trials increasing in difficulty (i.e., increasing the number of digits to be recalled). The test trials started at two-digit strings (e.g., 6 – 3), and increased up to nine digits for Forward and Sequencing, and eight digits for Backward (e.g., 9 – 4 – 3 – 7 – 6 – 2 – 1 – 8).

### Procedure

After providing written informed consent, participants completed the two speech perception tasks. Following the listening tasks, participants completed the battery of tasks assessing various cognitive processing capacities. The whole set of tasks was carried out within one experimental session, lasting ~60 min. All participants were tested individually in the same sound-attenuated testing room. The study was approved by the Human Research Ethics Advisory Panel (Psychology) at UNSW.

#### Speech perception

For both tasks, stimuli were presented and responses recorded using DMDX (Forster and Forster, 2003), a Windows-based display program with millisecond timer, on a desktop personal computer. Auditory stimuli were delivered to participants through Sennheiser HD 202 headphones.

For the auditory sentence verification task, each statement in this task were presented once to participants, in a randomized order within each part (foreign-accented and standard Australian). All participants heard the foreign-accented items first, and then items spoken in Standard Australian English. The practice items were presented prior to Part 1. Items were presented as soon as the participant had responded to the previous one, or after 5 s had elapsed with no response. Participants were instructed to respond as quickly but as accurately as they could, by pressing the right Shift key labeled “Yes” for true, and the left Shift key labeled “No” for false. Response times and error rates of decisions were recorded.

For the accented lexical decision task, all items were presented to participants in a randomized order, preceded by the practice items. Each item was presented once the participant had responded to the previous one or after 3 s had elapsed with no response. Participants were instructed to respond as quickly but as accurately as they could, by pressing the right Shift key labeled “Yes” for words, and the left Shift key labeled “No” for nonwords. Response times and error rates of decisions were recorded.

#### Executive functions

For the TMT, participants were instructed to connect the circles as quickly as possible without making mistakes while being timed for completion of each part. Mistakes were immediately corrected by the experimenter while the stopwatch was kept running. Completion time in seconds for each part was recorded. A ratio score (TMT-B/TMT-A) was calculated for each participant. The ratio score takes into account the baseline speed of performance without task switching in Part A, and is not affected by the starting performance in Part A.

Similarly for the CWIT, participants were instructed to complete each condition as quickly as possible without making mistakes. Completion time in seconds for each condition was recorded for each participant. Three contrast scores were calculated (see Delis et al., 2001): Inhibition Cost (i.e., Inhibition minus Color Naming), combined Inhibition/Switching Cost (i.e., Inhibition/Switching minus the sum of Color Naming and Word Reading), and Switching Cost (i.e., Inhibition/Switching minus Inhibition). The Inhibition Cost reflects the ability to inhibit the automatic tendency to read the written word in order to correctly name incongruent ink colors, while accounting for baseline speed of performance in naming color patches. The combined Inhibition/Switching Cost is a measure of both the ability to inhibit reading the word and task switch between naming colors and reading words, while accounting for baseline speed of performance in both naming color patches and reading words printed in black ink. The Switching Cost measures the ability to switch between naming incongruent ink colors and reading words, while partialing out performance in inhibition.

#### Information processing speed

On the Coding measure, participants were instructed to draw the symbols that matched with each number in order of appearance and without skipping any, and to do this as quickly as possible without making mistakes until told to stop. The number of correctly drawn symbols completed within a 2-min time limit was recorded for each participant, with a maximum total of 135. If the participant completed all items before the time limit expired, the completion time in seconds was recorded as well.

For Alphabet Backwards, participants were instructed to recite as quickly as they could without making mistakes. Errors were immediately corrected by the experimenter while the stopwatch was kept running. Completion time in seconds and the number of errors made were recorded. A performance index score (completion time/number correct) was calculated for each participant. This score reflects the average number of seconds required to obtain each correct answer, which provides an index of information processing speed taking into account performance accuracy (Williams et al., 1996).

#### Working memory

For Digit Span Forward, a sequence of digits was read out to participants who were required to recall the numbers in the same order, while Digit Span Backward required participants to recall the numbers in reverse order, and Digit Span Sequencing required participants to recall the numbers in ascending order (i.e., from smallest to largest). Each part was discontinued after the participant could not correctly recall two consecutive trials of the same length. One point was given for each correctly recalled sequence, with a maximum total of 48. The scores on each of the three parts were also recorded for each participant.

## Results

When analyzing the results of the sentence verification task, in addition to overall performance across all statements, the two types of statements (i.e., true and false) were examined separately, as they may produce different patterns of results. Specifically, false statements may provide less adequate contextual cues to help identify the sentences, given their often absurd meaning. Thus, listeners may need to rely more on cognitive resources when perceiving false statements than when listening to true statements. Table 1 presents the mean performance scores on each measure. It can be seen that the accented part in the sentence verification task produced significantly slower response times than the standard part. These differences were significant for true statements, false statements, and overall, *t*_(131)_ > 15, *p* < 0.001. However, participants made very few errors across both accented and standard sentences, and there were no significant differences across the two conditions, *t*_(131)_ < 1, *p* > 0.3. To examine whether cognitive processing measures predicted speech perception outcomes, analyses using simple regression were conducted. Each of the cognitive processing measures was used as a predictor variable in separate simple regression analyses, predicting performance on each of the speech perception measures (see Table 2 for correlations among the variables, and Table 3 for detailed regression results).

**Table 1:** **Performance scores on all measures**.

**Measure**	***M***	***SD***	**Range**	
			**Minimum**	**Maximum**
Sentence verification RT accented overall	2183.1	208.9	1886.0	3132.0
Sentence verification RT accented true statements	2190.9	214.1	1881.0	3033.0
Sentence verification RT accented false statements	2175.3	230.0	1802.0	3231.0
Sentence verification RT standard overall	1954.8	196.5	1641.0	3044.0
Sentence verification RT standard true statements	1942.4	205.1	1608.0	2963.0
Sentence verification RT standard false statements	1967.2	209.5	1656.0	3125.0
Sentence verification ER accented overall	1.7	2.8	0.0	10.0
Sentence verification ER accented true statements	1.3	3.8	0.0	20.0
Sentence verification ER accented false statements	2.1	4.1	0.0	12.5
Sentence verification ER standard overall	1.5	3.0	0.0	15.6
Sentence verification ER standard true statements	1.3	3.4	0.0	11.1
Sentence verification ER standard false statements	1.6	4.3	0.0	20.0
Accented lexical decision RT words	1080.8	88.1	790.0	1313.0
Accented lexical decision RT nonwords	1302.8	147.5	867.0	1646.0
Accented lexical decision ER words	9.7	5.3	0.0	28.2
Accented lexical decision ER nonwords	19.1	13.9	0.0	85.7
TMT Part A	22.7	7.7	10.9	55.9
TMT Part B	53.6	16.2	24.9	125.9
TMT ratio score (B/A)	2.5	0.8	1.0	5.3
CWIT color naming baseline	26.0	4.5	16.7	40.3
CWIT Word Reading baseline	20.2	3.2	13.2	31.5
CWIT Inhibition condition	42.5	8.4	26.2	66.5
CWIT inhibition/switching condition	51.2	9.0	36.0	84.4
CWIT inhibition cost	16.5	6.4	4.2	35.2
CWIT inhibition/switching cost	5.0	8.1	−17.4	30.1
CWIT switching cost	8.7	9.1	−16.6	36.2
Coding	86.1	14.2	54.0	135.0
Alphabet backwards completion time	64.0	48.2	5.8	380.5
Alphabet backwards index score (time/correct)	2.7	2.2	0.2	16.5
Digit span forward	11.1	2.5	6.0	16.0
Digit span backward	8.4	2.1	5.0	15.0
Digit span sequencing	7.8	1.7	5.0	15.0
Digit span total	27.3	4.9	19.0	42.0

**Table 2:** **Correlations among all variables**.

		**1**	**2**	**3**	**4**	**5**	**6**	**7**	**8**	**9**	**10**	**11**	**12**	**13**	**14**	**15**	**16**	**17**	**18**	**19**	**20**	**21**	**22**	**23**	**24**	**25**	**26**	**27**	**28**	**29**	**30**	**31**	**32**
1	Sentence verification RT accented overall	1.0																															
2	Sentence verification RT accented true statements	0.9^**^																															
3	Sentence verification RT accented false statements	0.9^**^	0.8^**^																														
4	Sentence verification RT standard overall	0.8^**^	0.8^**^	0.7^**^																													
5	Sentence verification RT standard true statements	0.7^**^	0.7^**^	0.6^**^	0.9^**^																												
6	Sentence verification RT standard false statements	0.8^**^	0.7^**^	0.7^**^	0.9^**^	0.8^**^																											
7	Sentence verification ER accented overall	0.1	0.2	0.1	0.1	0.1	0.1																										
8	Sentence verification ER accented true statements	0.2^*^	0.2^**^	0.1	0.2^**^	0.3^**^	0.2	0.7^**^																									
9	Sentence verification ER accented false statements	0.0	0.0	0.0	0.0	−0.1	0.0	0.7^**^	0.0																								
10	Sentence verification ER standard overall	0.1	0.0	0.1	0.1	0.1	0.2	0.0	0.1	−0.1																							
11	Sentence verification ER standard true statements	0.0	0.0	0.0	0.0	0.0	0.1	−0.1	0.0	−0.1	0.7^**^																						
12	Sentence verification ER standard false statements	0.1	0.1	0.1	0.2	0.2	0.2	0.0	0.2	−0.1	0.8^**^	0.2^*^																					
13	Accented lexical decision RT words	0.4^**^	0.4^**^	0.4^**^	0.5^**^	0.4^**^	0.5^**^	−0.1	0.0	−0.1	0.0	0.0	0.0																				
14	Accented lexical decision RT nonwords	0.5^**^	0.4^**^	0.5^**^	0.5^**^	0.5^**^	0.5^**^	0.0	0.1	0.0	0.0	0.0	0.0	0.7^**^																			
15	Accented lexical decision ER words	−0.2	−0.1	−0.2^*^	−0.2^*^	−0.2^*^	−0.1	0.0	0.0	0.0	0.1	0.1	0.1	−0.2^*^	−0.4^**^																		
16	Accented lexical decision ER nonwords	0.2^*^	0.2^*^	0.2^*^	0.2^**^	0.2^*^	0.2^*^	−0.1	0.0	−0.1	0.1	0.0	0.2	0.1	0.4^**^	−0.3^**^																	
17	TMT Part A	0.2^*^	0.2^*^	0.2	0.2^*^	0.2	0.2^*^	0.1	0.0	0.1	0.0	0.1	−0.1	0.1	0.0	0.0	0.0																
18	TMT Part B	0.2^**^	0.2^*^	0.3^**^	0.2^*^	0.2^*^	0.2^*^	0.1	0.1	0.1	0.0	−0.1	0.1	0.0	0.1	0.1	0.0	0.4^**^															
19	TMT ratio score (B/A)	0.0	0.0	0.0	−0.1	0.0	−0.1	0.1	0.1	0.1	0.1	−0.1	0.2^*^	−0.1	0.0	0.1	0.0	(−0.5^**^)^#^	(0.5^**^)^#^														
20	CWIT color naming baseline	0.3^**^	0.3^**^	0.3^**^	0.3^**^	0.3^**^	0.2^**^	0.0	0.1	−0.1	0.0	−0.1	0.1	0.2^*^	0.2^**^	0.0	0.1	0.2^**^	0.4^**^	0.1													
21	CWIT Word Reading baseline	0.3^**^	0.2^*^	0.3^**^	0.2^*^	0.2^*^	0.2^*^	0.0	0.0	0.0	0.0	−0.1	0.1	0.1	0.1	0.0	0.1	0.2^*^	0.3^**^	0.1	0.7^**^												
22	CWIT Inhibition condition	0.2^*^	0.2^*^	0.2^*^	0.2^*^	0.2^*^	0.2^*^	0.0	0.1	−0.1	0.2	0.0	0.2^*^	0.2^*^	0.2^*^	0.0	0.2^*^	0.2^*^	0.3^**^	0.0	0.7^**^	0.5^**^											
23	CWIT inhibition/switching condition	0.3^**^	0.2^*^	0.3^**^	0.3^**^	0.3^**^	0.2^**^	0.1	0.1	0.1	0.1	−0.2	0.2^**^	0.1	0.1	0.1	0.3^**^	0.2^*^	0.5^**^	0.2^*^	0.4^**^	5^**^	0.5^**^										
24	CWIT inhibition cost	0.1	0.1	0.1	0.1	0.1	0.1	0.0	0.0	−0.1	0.2^*^	0.1	0.2^*^	0.1	0.1	0.0	0.2	0.1	0.1	0.0	(0.2)^#^	0.2	(0.8^**^)^#^	0.3^**^									
25	CWIT inhibition/switching cost	0.0	0.0	0.1	0.1	0.1	0.1	0.2	0.1	0.1	0.1	−0.1	0.2	−0.1	0.0	0.1	0.2^*^	0.0	0.2	0.1	(−0.3^**^)^#^	(−0.2^*^)^#^	−0.1	(0.7^**^)^#^	0.2								
26	CWIT switching cost	0.1	0.0	0.1	0.1	0.1	0.1	0.2	0.1	0.2	−0.1	−0.2	0.0	−0.1	0.0	0.1	0.1	0.0	0.2^*^	0.2^*^	−0.2^*^	0.1	(−0.5^**^)^#^	(0.6^**^)^#^	−0.5^**^	0.7^**^							
27	Coding	−0.2^**^	−0.2^**^	−0.2^*^	−0.2^*^	−0.2^*^	−0.2^*^	0.0	−0.1	0.1	0.0	0.0	0.0	−0.2^*^	−0.2^*^	−0.1	−0.1	−0.4^**^	−0.4^**^	0.0	−0.4^**^	−0.3^**^	−0.4^**^	−0.4^**^	−0.3^**^	−0.1	0.0						
28	Alphabet backwards completion time	0.1	0.1	0.1	0.1	0.1	0.1	0.1	0.1	0.1	0.0	−0.1	0.1	0.1	0.1	0.1	0.0	0.1	0.4^**^	0.2^**^	0.4^**^	0.3^**^	0.3^**^	0.3^**^	0.2^*^	0.0	0.0	−0.3^**^					
29	Alphabet backwards index score (time/correct)	0.1	0.1	0.1	0.1	0.1	0.0	0.1	0.0	0.1	0.0	−0.1	0.1	0.1	0.1	0.1	0.0	0.1	0.4^**^	0.2^**^	0.3^**^	0.3^**^	0.3^**^	0.3^**^	0.2^*^	0.0	0.0	−0.2^**^	1.0^**^				
30	Digit Span Forward	0.0	0.0	0.0	0.1	0.1	0.1	−0.2	−0.2	−0.1	0.0	0.1	−0.1	0.1	0.0	−0.1	0.0	−0.1	−0.2^**^	−0.2^*^	−0.2^**^	−0.2^**^	−0.2^**^	−0.2^*^	−0.2	0.0	0.1	0.1	−0.4^**^	−0.3^**^			
31	Digit span backward	−0.1	−0.1	−0.2	−0.1	−0.1	−0.1	0.0	−0.1	0.1	0.0	0.1	−0.1	0.0	0.0	−0.1	−0.1	−0.1	−0.2^**^	−0.2	−0.3^**^	−0.3^**^	−0.3^**^	−0.2^*^	−0.2	0.1	0.1	0.1	−0.3^**^	−0.3^**^	0.4^**^		
32	Digit span sequencing	−0.1	−0.1	−0.1	0.0	−0.1	0.0	0.1	0.0	0.2^*^	0.0	0.0	0.0	0.0	0.0	−0.1	−0.1	−0.3^**^	−0.4^**^	−0.1	−0.3^**^	−0.2^*^	−0.3^**^	−0.2^*^	−0.2^*^	0.0	0.1	0.2^*^	−0.3^**^	−0.3^**^	0.3^**^	0.5^**^	
33	Digit span total	−0.1	−0.1	−0.1	0.0	0.0	0.0	0.0	−0.1	0.1	0.0	0.0	−0.1	0.1	0.0	−0.1	−0.1	−0.2	−0.3^**^	−0.2^*^	−0.4^**^	−0.3^**^	−0.3^**^	−0.2^**^	−0.2^*^	0.0	0.1	0.2	−0.4^**^	−0.4^**^	0.8^**^	0.8^**^	0.7^**^

* *Significant correlation, p < 0.05*.

** *Significant correlation, p < 0.01*.

# *Circular correlation (one variable subtracted or divided from the other)*.

**Table 3:** **Simple regressions predicting each speech perception measure using each cognitive processing measure separately (significant regressions only)**.

**Criterion variable**				
** Predictor variable**	***b***	***t***	***p***	***r^2^***
**SENTENCE VERIFICATION RT ACCENTED OVERALL**				
TMT Part B	3.11	2.81	0.006	0.058
CWIT inhibition condition	5.20	2.44	0.016	0.044
CWIT inhibition/switching condition	6.11	3.12	0.002	0.071
Coding	−3.58	−2.86	0.005	0.060
**SENTENCE VERIFICATION RT ACCENTED TRUE STATEMENTS**				
TMT Part B	2.66	2.33	0.022	0.041
CWIT Inhibition condition	5.42	2.48	0.014	0.046
CWIT inhibition/switching condition	4.85	2.38	0.019	0.042
Coding	−3.67	−2.86	0.005	0.060
**SENTENCE VERIFICATION RT ACCENTED FALSE STATEMENTS**				
TMT Part B	3.55	2.93	0.004	0.063
CWIT inhibition condition	4.98	2.11	0.037	0.034
CWIT inhibition/switching condition	7.38	3.45	0.001	0.085
Coding	−3.49	−2.51	0.013	0.047
**SENTENCE VERIFICATION RT STANDARD OVERALL**				
TMT Part B	2.42	2.30	0.023	0.040
CWIT inhibition condition	5.06	2.53	0.013	0.048
CWIT inhibition/switching condition	5.86	3.19	0.002	0.074
Coding	−2.94	−2.48	0.015	0.046
**SENTENCE VERIFICATION RT STANDARD TRUE STATEMENTS**				
TMT Part B	2.41	2.19	0.030	0.036
CWIT inhibition condition	5.31	2.54	0.012	0.048
CWIT inhibition/switching condition	6.01	3.13	0.002	0.071
Coding	−2.68	−2.15	0.033	0.035
**SENTENCE VERIFICATION RT STANDARD FALSE STATEMENTS**				
TMT Part B	2.43	2.17	0.032	0.035
CWIT inhibition condition	4.81	2.24	0.027	0.038
CWIT inhibition/switching condition	5.72	2.89	0.004	0.061
Coding	−3.20	−2.53	0.013	0.048
**SENTENCE VERIFICATION ER ACCENTED OVERALL**				
None				
**SENTENCE VERIFICATION ER ACCENTED TRUE STATEMENTS**				
None				
**SENTENCE VERIFICATION ER ACCENTED FALSE STATEMENTS**				
Digit span sequencing	0.43	1.98	0.049	0.030
**SENTENCE VERIFICATION ER STANDARD OVERALL**				
CWIT inhibition cost	0.09	2.11	0.037	0.034
**SENTENCE VERIFICATION ER STANDARD TRUE STATEMENTS**				
None				
**SENTENCE VERIFICATION ER STANDARD FALSE STATEMENTS**				
TMT ratio score (B/A)	1.15	2.46	0.015	0.045
CWIT inhibition condition	0.11	2.51	0.013	0.047
CWIT inhibition/switching condition	0.11	2.81	0.006	0.058
CWIT inhibition Cost	0.13	2.29	0.024	0.039
**ACCENTED LEXICAL DECISION RT WORDS**				
CWIT inhibition condition	2.23	2.49	.014	0.045
Coding	−1.23	−2.30	.023	0.039
**ACCENTED LEXICAL DECISION RT NONWORDS**				
CWIT inhibition condition	3.45	2.29	0.024	0.039
Coding	−1.82	−2.03	0.044	0.031
**ACCENTED LEXICAL DECISION ER WORDS**				
None				
**ACCENTED LEXICAL DECISION ER NONWORDS**				
CWIT inhibition condition	0.29	2.06	0.041	0.032
CWIT inhibition/switching condition	0.39	3.00	0.003	0.065
CWIT inhibition/switching cost	0.32	2.14	0.034	0.034

For auditory sentence verification performance, TMT Part B, CWIT Inhibition and Inhibition/Switching conditions, and Coding each significantly predicted overall response times for the accented part, *t*_(130)_ > |2.4|, *p* < 0.02, and overall response times for the standard part, *t*_(130)_ > |2.3|, *p* < 0.03. These four variables also predicted response times for both true statements, *t*_(130)_ > |2.3|, *p* < 0.03, and false statements, *t*_(130)_ > |2.1|, *p* < 0.04, within the accented part, and both true statements, *t*_(130)_ > |2.1|, *p* < 0.04, and false statements, *t*_(130)_ > |2.1|, *p* < 0.04, within the standard part. That is, better performance on each of those measures (i.e., faster completion times for TMT or CWIT, or higher Coding scores) led to faster response times for both accented and standard sentence verification. For the measure of error rates, Digit Span Sequencing was a significant predictor for false statements in the accented part, *b* = 0.43, *t*_(130)_ = 1.98, *p* = 0.049, where higher scores led to higher error rates. For the standard part, CWIT Inhibition Cost (Inhibition—Color Naming) was a significant predictor of overall error rates, *b* = 0.09, *t*_(130)_ = 2.11, *p* = 0.037, with better performance predicting fewer errors, while TMT ratio score, CWIT Inhibition condition, Inhibition/Switching condition, and Inhibition Cost each significantly predicted error rates for non-foreign-accented false statements, *t*_(130)_ > |2.2|, *p* < 0.03.

For accented lexical decision, CWIT Inhibition and Coding were significant predictors of response times for both words, *t*_(130)_ ≥ |2.3|, *p* < 0.03, and nonwords, *t*_(130)_ > |2.0|, *p* < 0.05. That is, better performance on each of those measures led to faster response times. For error rates, CWIT Inhibition condition, Inhibition/Switching condition, and combined Inhibition/Switching Cost (Inhibition/Switching—combined Color Naming and Word Reading) each significantly predicted error rates for nonwords, *t*_(130)_ > |2.0|, *p* < 0.05, with better performance leading to fewer errors.

In summary, better performance on some measures of executive function, including TMT Part B, CWIT Inhibition and CWIT Inhibition/Switching, and better performance on the Coding measure of information processing speed predicted faster response times for both versions of the auditory sentence verification task (accented and non-foreign-accented). Better executive function performance, as indicated by CWIT Inhibition, and better performance on the Coding measure of information processing speed also predicted faster response times for the accented lexical decision task. For errors, better performance on inhibition and switching measures, particularly those in the CWIT, predicted lower error rates for non-foreign-accented sentence verification, especially for false statements, as well as lower error rates for nonwords in the accented lexical decision task. There were, however, some differences in the executive function measures that predicted response times vs. those that predicted error rates. Specifically, those that predicted error rates included measures where baseline performance has been partialled out (i.e., TMT ratio score, CWIT Inhibition Cost, and combined Inhibition/Switching Cost), whereas those that predicted response times did not (i.e., TMT Part B, CWIT Inhibition, and CWIT Inhibition/Switching). Lastly, working memory did not consistently predict performance on the speech perception tasks.

## Discussion

The present study investigated speech perception as a function of more general cognitive processing performance, so that potential influences of these cognitive processing capacities on speech perception could be examined. Specifically, the influences of executive functions (both switching and inhibition), information processing speed, and working memory were assessed. Previous findings with older adults have shown that executive function processes, specifically task switching as measured by the TMT, predict performance in perception of unfamiliar accented speech, while information processing speed, as measured by the Coding test, did not (Adank and Janse, 2010).

In the present study with young adults, both executive functioning and information processing speed were found to be associated with speech perception performance. Task switching, inhibitory control, and information processing speed predicted response times for both accented and standard sentence stimuli. Inhibition and information processing speed also predicted speed of responding to accented word-length stimuli. However, the measures of executive functions that predicted speech perception response times were those that did not adjust for baseline performance. Since those indicators were raw completion times on the relevant tasks (i.e., TMT Part B, CWIT Inhibition condition, and Inhibition/Switching condition), they may also reflect processing speed to some degree, rather than being pure measures of executive function processes. The influences of information processing speed on speech perception found in the present study is likely due to the nature of the speech perception tasks, which required participants to make speeded decisions and responses. The speech perception task in the study by Adank and Janse (2010) did not measure response speed, but instead measured the number of words correctly repeated after each sentence, which allowed participants to take their time in making responses.

When looking at the accuracy measure of the speech perception tasks, only executive function processes predicted performance, and information processing speed did not, consistent with Adank and Janse (2010). However, the influence of executive functions on speech perception accuracy in the present study was found only for some types of stimuli, namely false statements and nonwords. Further, unlike for speech perception response times, the measures that predicted speech perception accuracy included those that adjusted for baseline performance (i.e., TMT ratio score, CWIT Inhibition Cost, and combined Inhibition/Switching Cost), which may be more rigorous and pure indicators of executive functioning. The differences between the outcomes of the present study and those of Adank and Janse (2010) may be due to differences in functioning between young and older adults. Older adults were shown to have considerably greater difficulty understanding unfamiliar accented speech, as well as a lesser degree of perceptual adaptation following exposure, compared to young adults (Adank and Janse, 2010). Older adults may, therefore, need to draw on cognitive resources to a greater extent than young adults to perceive unfamiliar accented speech (Pichora-Fuller, 2003; Gordon-Salant and Fitzgibbons, 2004). Young adults may only need to draw on cognitive resources for items like false statements and nonwords, which do not provide adequate contextual cues to be easily perceived.

The present results are partly consistent with those of Baese-Berk et al. (2015), who found that switching predicted accented speech recognition, but inhibition did not. Compared to the study by Baese-Berk et al. (2015), the accents in the present study were less familiar to participants, and thus may require increased cognitive resources for successful perception. Specifically, the input signal of highly unfamiliar accents may differ more from expectations or stored representations than accents that are more familiar. Listeners may, therefore, need to utilize inhibitory control to a greater extent to match the incoming deviant signal to the correct representations, that is, by inhibiting the activation of multiple competing representations that are also potential matches for the input signal (Gordon-Salant and Fitzgibbons, 2004; Rudner and Lunner, 2014; Rönnberg et al., 2016).

The effortful task in the perception of nonstandard speech is overcoming mismatches between perceived phonological input and stored phonological representations, in order to comprehend the intended meaning. As such, numerous cognitive processes may be required for successful comprehension, that is, not only aspects such as inhibition, switching, and information processing speed, processes like working memory may also need to be drawn upon (Janse and Adank, 2012; Rönnberg et al., 2013). The ELU model (Rönnberg, 2003; Rönnberg et al., 2008, 2013) explicates that working memory plays a role in speech perception, under varying listening conditions, and by listeners with or without hearing impairments. In the present study, however, working memory was not found to predict perception of accented and standard speech, by young, healthy listeners. Previous research has suggested that working memory capacity may serve as a limiting factor when perceiving speech with reduced intelligibility, such as unfamiliar accented speech (e.g., Janse and Adank, 2012; see also Rönnberg et al., 2013). It is possible that working memory only plays a role when perceiving speech with severely reduced intelligibility. Indeed, evidence for an impact of working memory on speech perception has typically been found using low intelligibility speech or speech that may initially be unintelligible, such as speech-in-noise and synthetic speech (e.g., Francis and Nusbaum, 2009; Janse and Adank, 2012; see Akeroyd, 2008, for a review). Furthermore, it has been suggested that simple span tasks, such as digit span, mainly assess storage capacities in short-term memory, which may not be a good predictor of language comprehension, and that *complex working memory capacity* is the ability involved in understanding language (Rönnberg et al., 2013). Although, the Digit Span task used in the present study had two of three parts that entailed simultaneous storage and processing of information, a more multifaceted task may be required to assess complex working memory capacity, for example reading span and other complex span (C-span) tasks, dual n-back and recall n-back tasks, or binding tasks (see e.g., Rönnberg et al., 2013; Wilhelm et al., 2013). Such tasks require continuous mental monitoring and updating, in addition to simultaneous storage and manipulation. Finally, not all studies have found an influence of working memory on speech processing, particularly for young listeners with normal hearing. In a meta-analysis, Füllgrabe and Rosen (2016) found that working memory (as measured by the reading span task) was not consistently involved in speech perception under adverse listening conditions (i.e., comprehending speech-in-noise), for young, healthy listeners.

To conclude, the present study showed that cognitive factors play a role in determining perception of variable speech in healthy young adults. In particular, aspects of executive function predicted accuracy of perception, while both information processing speed and executive functioning predicted speed of performance in the perception tasks. These influences were observed with both sentence and word-length stimuli. Furthermore, accented speech perception was not likely to be influenced by relative familiarity with any particular accent, as multiple unfamiliar accents were included in the stimulus set. The results of the present study suggest that stronger abilities in cognitive switching and inhibition are likely to be helpful for matching unfamiliar pronunciations of speech sounds to stored representations, for example by being able to inhibit incorrect competing representations and shifting to other possible matches (Gordon-Salant and Fitzgibbons, 2004; Adank and Janse, 2010; Rudner and Lunner, 2014; Rönnberg et al., 2016). Additionally, information processing speed likely affects the ability to rapidly learn the regularities in the way speech sounds are produced, thus affecting the speed of responding to speech stimuli (Pichora-Fuller, 2003; Adank and Janse, 2010). There is scope for future work to further examine the involvement of various cognitive capacities on speech perception under different types of adverse listening conditions, for example increased difficulty in the listening conditions posed by environmental factors, such as background noise, over and above the unfamiliar foreign accent.

## Author contributions

Conception or design of the work: LT, MT. Data collection: LT. Data analysis and interpretation: LT, MT. Drafting the work: LT. Critical revision of the work: LT, MT. Final approval of the work to be published: LT, MT.

## Funding

This research was supported by an Australian Postgraduate Award from the Australian Government awarded to LT.

### Conflict of interest statement

The authors declare that the research was conducted in the absence of any commercial or financial relationships that could be construed as a potential conflict of interest.
