# Supplementary material for: Influences of Cognitive Processing Capacities on Speech Perception in Young Adults
Source: Front Psychol. 2017 Feb 24;8:266. doi: 10.3389/fpsyg.2017.00266 (PMC5323404; doi:10.3389/fpsyg.2017.00266)
Supplement: Supplementary file 1 [file DataSheet1.docx]

**Appendix A**

**Items in the Auditory Sentence Verification Task**

**Practice items:**

Rats have teeth.

Nuns are made in factories.

Ants are living creatures.

Tractors grow in gardens.

Desks wear clothes.

Fishes live under the water.

**Accented test items:**

A truck can fly.

Clouds have roots in the ground.

Cows have wings.

Dogs have four ears.

Plants grow in the sky.

Stones are usually soft.

Trees can bite you.

You drink flies when you are thirsty.

You need your ears to eat.

Your nose is a part of your leg.

Babies sometimes cry.

Books have pages.

Fire is hot.

Most people have two legs.

People wear shoes on their feet.

The sun shines during the day.

Water is wet.

You need heat to cook things.

You see with your eyes.

You usually sleep with your eyes closed.

**Standard test items:**

Bananas ride bicycles.

Cars have feet.

Cats can lay eggs.

Houses have two legs.

It is nice to eat shoes.

Milk comes from chickens.

Oranges have ears.

People have two noses.

The sun is cold.

Your fingers are joined on to your feet.

Bicycles have wheels.

Birds have feathers.

Clouds are in the sky.

Dogs can bark.

Growing plants need water.

You can eat food.

You can wear a hat on your head.

You need your tongue for talking.

You sweat when you are hot.

Your ears are attached to your head

**Appendix B**

**Items in the Accented Lexical Decision Task**

**Practice items:**

**Words**

broad

plain

root

seat

source

hurry

immediately

powerful

rubber

scientist

**Nonwords**

palt

ruch

sheece

tuce

wilp

brotash

cumia

dowuble

rehune

trederary

**Test items:**

**Words**

bell

clock

dust

fence

flow

guide

height

lake

mass

nest

peace

range

score

shop

smooth

throw

tone

track

view

wave

atmosphere

belong

chapter

community

continue

dangerous

discover

favourite

flower

generally

handle

instance

master

observe

ordinary

popular

silent

solution

structure

support

**Nonwords**

bish

chusk

crawn

dasp

ferk

floin

gret

hoft

lurt

molk

nooth

plource

roax

slint

spoag

talf

thast

thid

vact

wrenth

altuary

baklara

cegalia

cliney

dybical

esinent

flegulity

flubry

fruantry

junger

kesom

lemusal

mutek

omsify

orsel

pladul

sinky

smennial

twidal

wetry
